# The Impact of Negative Affectivity on Teacher Burnout

**DOI:** 10.3390/ijerph182413124

**Published:** 2021-12-13

**Authors:** Philippe A. Genoud, Elisabeth L. Waroux

**Affiliations:** Department of Education, University of Fribourg, 1700 Fribourg, Switzerland; elisabeth.waroux@unifr.ch

**Keywords:** negative affectivity, personality, burnout

## Abstract

Teachers’ well-being, including burnout, impacts the stress and well-being of students. Understanding the development of burnout requires not only an examination of stressors, but also a consideration of personality factors. While teachers are subject to many pressures in their profession, they have personalities that make them more or less vulnerable. Our research with 470 secondary school teachers reveals four distinct negative affectivity profiles. Our results show that negative affectivity (tendency to feel depression, anxiety, or stress) plays a role in the development of burnout. However, while teachers with a more anxious profile experience greater emotional exhaustion, those with a depressive profile have more difficulty developing a strong sense of personal accomplishment. The findings highlight the need to take into account the various facets of negative affectivity, particularly in order to be able to propose prevention and intervention approaches adapted to these specific profiles.

## 1. Introduction

Burnout is defined as the response to chronic interpersonal stressors and is generally structured with three main components: emotional exhaustion, feelings of cynicism and detachment (depersonalization), and a lack of personal accomplishment [1] and is often assessed through the Maslach Burnout Inventory (MBI) [2,3]. Professional burnout concerns all types of activities, but in particular the ‘caring’ professions (e.g., nursing) and, overall, professions characterized by demanding interactions with service recipients such as teaching [4]. Indeed, teachers are under a lot of stress in their classrooms, but the pressure also comes from outside as, for example, increased administrative workload or conflictual relations with pupils’ parents [5].

Burnout’s three dimensions are linked to different processes. Schaufeli and Dierendonck [6] call emotional exhaustion the affective component. It is characterized by feeling that one’s emotional resources are depleted and that one has no energy left. Regarding depersonalization, they speak of the attitudinal component. Depersonalization is characterized by being less social and sometimes even cynical. In the school setting, this detachment can be towards students or colleagues alike [7]. Diminished personal accomplishment is the cognitive component of the model. Here, it is a self-representation as someone who cannot face work pressures, which is accompanied by guilt.

How the different parts of burnout interact with each other is controversial [8,9]. Regarding teacher burnout, Friedman [10] highlights two distinct causal patterns leading the development of this syndrome in his Multiple Pathway to Burnout (MPB): (a) a cognitive track resulting from a strong sense of professional non-accomplishment and that is found in particular in very idealistic teachers; and (b) an emotional track related with a feeling of overload facing too many stressors. Thus, the etiology of burnout is not the same in every teacher, since personality, but also personal background, can explain individual differences [11].

Teacher burnout has an impact on students’ well-being. In their study on stress contagion, Oberle and Shonert-Reichl [12] show that students in burnt-out teachers’ classrooms had significantly higher cortisol levels than their peers in classes with non-burnt-out teachers. Furthermore, more negative school environments have a negative impact on children’s mental health [13]. Therefore, understanding what impacts teachers’ mental health is important in order to preserve children as much as possible. Unlike in other professions with high burnout rates, teachers have a prolonged contact with students which makes their well-being of paramount importance as they interact with children and adolescents who are still building their identity.

In understanding the mechanisms that bring a teacher to feel professionally exhausted, one can categorize different specific factors [14,15]. First, there are those linked to one’s professional context. Although there is little research conducted comparing burnout levels of different professions, the most common samples these last 30 years tested helping professions and professions with an important “client” contact component [4,16]. Therefore, burnout among nurses, teachers and social workers has been thoroughly investigated, as has been burnout among police officers. These professions share important stress generated by repeated social contacts. However, one should remember that personality factors can also explain both career choices and stress response.

Studies are not limited to these careers. By expanding the concept of burnout to other types of activities, researchers were able to show that it can happen in any activity or career. For example, burnout was observed in managers in the financial sector [17], athletes [18], parents [19] and students [20]. Indeed, one of the most prevalent factors in developing burnout is a significant and regular stress which is very prevalent in these situations even though, sometimes, the “client” interactions do not exist. The social context (sometimes linked to the professional one) also has an impact on developing burnout. Several researchers have shown that social support (including from one’s management) can buffer and delay the onset of burnout (e.g., [21,22]).

However, all of these contextual clues do not explain the onset of burnout. Individual characteristics (gender, self-esteem, emotional competence or personality traits) partially explain individual differences [23]. For example, Schaufeli and Enzmann [24] found over one hundred publications examining personalities’ (or linked variables) influence on burnout. Indeed, when faced with similar pressures, not all teachers will end up in burnout. Professionals in similar contexts do not react in the same way to stress factors [16] and personality can be the catalyst in the links between stress and burnout.

Many studies in different professional contexts have looked at the link between personality factors and burnout levels. Although pioneering studies are generally focused on teachers, police officers and medical personnel, current publications also examine a larger group of careers in varying sectors. One must be careful in interpreting results in transversal studies due to overestimation of some coefficients [25] as well as in comparing conclusions in studies that sometimes use very different measurement tools [26]. However, personality traits’ impact is largely agreed upon [27]. More specifically, neuroticism seems to strongly predict the onset of burnout in workers [28,29,30,31,32].

Neuroticism is a normal personality trait (non-pathological) that impacts some people more than others when it comes to feeling negative affects and refers to aspects such as anger, anxiety, depression, self-consciousness, impulsiveness and irritability [33,34]. This personality trait underscores emotional instability, perhaps even a vulnerability, which can affect ones’ behavior as emotions can interfere with adaptative capabilities in different daily situations. In comparing different contextual factors and neuroticism, Bianchi [35] underscores the weight of personality in comparison with professional pressure or job support. Whilst a lot of the research on burnout aims to better understand the impact of different stressors and to develop adaptive coping strategies in workers [5,11], these results underline the importance of personality traits and that these should not be minimized. Depending on one’s personal way of dealing with work and personal pressures, one will feel emotionally depleted more or less quickly.

Among other personality traits, Bhowmick and Mulla [36] show, for example, that agreeableness and conscientiousness predict the personal accomplishment dimension in burnout. The link between neuroticism and emotional exhaustion is weak in this study which is surprising and could be explained by the sample of police officers used. However, low scores of agreeableness and conscientiousness are also associated with high levels of parental burnout alongside neuroticism [37]. Armon, Shirom, and Melamed [38] also add conscientiousness to the influence of neuroticism. Introversion also seems to be a factor that predisposes to burnout [39]. Indeed, in another study, Cano-García, Padilla-Muñoz, and Carrasco-Ortiz [40] show the impact of introversion whereas agreeableness appears to be a protective trait (moderating variable). In the end, however, neuroticism is the personality trait shown as having the most impact on emotional exhaustion.

Sosnowska, De Fruyt, and Hofmans [41] go one step further in that direction by approaching personality measures in a dynamic way. In order to evaluate neuroticism, these authors distinguish in their measurement this personality trait’s baseline level, variability over time and the attractor strength. The attractor strength is the speed at which a person returns to his or her baseline level after a temporary variation. They showed that individuals with high baselines and high variations experience more burnout. Their results also show an interaction effect where people with high attractor strength have high burnout rates only if their baseline is also high.

Furthermore, one should note that although slight variations in emotional instability can be seen over time, neuroticism is a personality trait which changes very little over the course of one’s adult life. This is why Varghese et al. [42] looked to find moderating factors to lessen the impact of personality on the onset of burnout. These authors specifically show that psycho-social and professional support given by mentors to the most fragile employees is an efficient buffer to burnout.

Results from the many studies on personality traits (and those on neuroticism in particular) are complementing each other. However, they systematically incorporate these personality traits without distinguish their facets. This dimensional structure of personality is particularly important to consider to better understand how the person functions, especially when it comes to disorders’ etiologies [43]. Indeed, neuroticism regroups several more specific factors [33] which need to be discussed individually in order to highlight the heterogeneity of participants who have a high score on this dimension. Therefore, it seems important to dig deeper into the link between personality and burnout by looking more closely at the impact of this negative affectivity’s three components.

### Negative Affectivity as Predictor of Burnout

The different subscales of negative affectivity (depression, anxiety and stress) have specific characteristics linked to them. These personality traits are akin to the corresponding disorders (see DSM) without being pathological. We will go further into each one below.

Depression has similar physical and psychological effects as burnout and the overlap is substantial [44]. It is characterized by sleeping difficulties, loss of energy, low self-esteem, and diminished social interactions. Although an important overlap Bianchi, Schonfeld, and Verkuilen [45] point out that each disorder also has specific characteristics. Indeed, some researchers see burnout and depression as two distinct issues [24]. Maslach, Schaufeli, and Leiter [46] distinguish them by the fact that burnout is linked to one’s professional activities whereas depression does not have a specific focus and can happen in different settings. Tavella and Parker [47] have specifically highlighted differences in the experiences for participants having had both pathologies. These recent results, highlighted by a qualitative methodology this time, suggest that although burnout and depression are similar in several aspects, they belong in two separate processes. We should note that our research’s goal is not to investigate the already well-researched link between depression as a pathology and burnout [48]. Rather, it is to understand how having more negative affects (in a non-clinical population) impacts the onset of burnout. According to Ciobanu et al. [49], “the Depression scale items assess symptoms related to dysphoria, hopelessness, devaluation of life, self-deprecation, lack of interest, anhedonia, and inertia”, without aiming for pathological criteria.

Anxiety is also linked to burnout. However, research has focused more on the corresponding personality trait than on the disorder. Turnipseed [50] shows significant correlations between burnout and the anxiety-trait measure of Spielberger’s inventory [51]. It may be that this emotional vulnerability makes some people more prone to developing burnout, starting a vicious cycle as anxiety is also a by-product of burnout. In fact, cognitive appraisal (and more specifically threat perception) has a moderating effect on the link between the anxiety trait and burnout [18].

Regarding the anxiety personality trait as part of negative affectivity, it is assessed by the Anxiety subscale items. The items include “physiological arousal (e.g., cardiac rate, breathing difficulty, and body temperature), situational anxiety, and subjective experience of anxious affect (e.g., fear and panic attacks)” [49]. This dimension clearly aligns in a neuroticism trait without altering social functioning.

The link between stress and burnout has been thoroughly documented in scientific literature, in particular for teachers whose sources of stress include students, their parents and sometimes their school’s administration [5]. It is largely accepted that an important and prolonged stress may cause burnout in individuals who do not have the resources to cope with these external pressures. This causal effect was particularly well exposed in Selye’s [52] general adaptation syndrome model. It presents three phases after one’s exposure to a stressor: alarm reaction that mobilizes resources, resistance to cope with stressors, and exhaustion when reserves are depleted. Stress, therefore, comes from the differences between environmental demands and personal resources. However, one should note that when measuring negative affectivity, “stress” is not the presence of stressors but rather a heightened stress reactivity. Indeed, the stress subscale measures the individual’s feelings of stress and ability to cope with pressure rather than stressors themselves. It includes “items assessing difficulty in relaxing, nervous arousal, tension, and irritability” [49].

Concretely (and without getting into the pathological dimensions associated with these personality traits) our first objective is to try highlighting negative affectivity patterns teachers may have using cluster analysis. The link between burnout, anxiety and depression (under a pathological angle) is already well documented (e.g., [48]), a detailed examination of personality factors should allow for a better understanding of the etiology of burnout. Indeed, different authors underscore how necessary it is to consider these dimensions together as profiles rather than individually (e.g., [27]). This is what, among others, Martinez-Monteagudo et al. [53] do by identifying different emotional intelligence profiles which are then put in relation with burnout dimensions. The second objective is to determine whether these profiles lead to different burnout levels, in particular taking account burnout’s three-dimensionality. Although we make a theoretical link between personality and burnout, one should note that we do not infer causality between the two.

## 2. Materials and Methods

### 2.1. Participants

In Switzerland, education is a responsibility of the canton (State or province). The French-speaking cantons have decided harmonize their systems while still keeping their regional autonomy and responsibilities. Fribourg is a bilingual canton and therefore has two school systems (French-speaking and German-speaking). The link to the online questionnaire was sent to all of the secondary I (grades 9–11) teachers in Fribourg’s French-speaking school system (14 schools). We received answers from 35.8% of teachers. Participants in this research are teachers (N = 470) between 24 and 63 years of age (M = 40.3; SD = 10.3) working in the last three years of compulsory schooling. In terms of gender, the sample seems representative of the population for theses degrees (38% male and 62% female; six teachers did not respond to the gender question). Participants are equally spread among the school grades (9, 10 and 11), with 43% of them teaching all these three grades. The distribution between the tracks (class ability level) is equally well balanced (with 45% of them teaching in all three tracks). Participants teach a variety of subject and most of them teach two to three subjects.

The research was approved by the *Département de l’instruction, de la culture et du sport* (Department of schooling, culture and sports) which reviews the appropriateness and ethics of any research conducted in schools; all participants (respectively their legal representatives) sign consent forms. In order to guarantee anonymity, the authors were asked to have as few socio-demographic and context questions as possible (e.g., marital status, family status, etc.). Indeed, due to the nature of the population, cross-referencing this data would allow for the easy identification of the participants. All filled-in questionnaires were included in the study, without any exclusions.

### 2.2. Tools

While Maslach’s original burnout scale [2] included twenty-two items on a seven-point scale (from never to every day), the questionnaire used in our study, based on the version validated by Dion and Tessier [54], includes twenty-seven items. It measures emotional exhaustion, depersonalization and lack of personal accomplishment. Several items were doubled to consider the impact of colleagues and students (e.g., “Don’t really care what happens to pupils”, and “Don’t really care what happens to colleagues”). Internal consistency (compared [54]) is acceptable to good: emotional exhaustion 0.89 (0.90); depersonalization 0.69 (0.64), and lack of personal accomplishment 0.77 (0.74).

In order to assess negative affectivity, the French translation of the Depression Anxiety Stress Scales (DASS) by Lovibond and Lovibond [55] was used. The translation was validated by Ciobanu et al. [49]. It consists of a 42-item questionnaire (14 items for each scale) where participants self-report negative states. According to Ciobanu et al. [49], items were selected to assess the particular characteristics of these personality traits, while ensuring that each item assessed only one of the three dimensions. The reliability analysis was slightly lower in our sample than in the validation one but the coefficients show a strong homogeneity of items: depression 0.87 (0.94), anxiety 0.82 (0.88), and stress 0.85 (0.91).

Whether all three dimensions are independent from one another is, of course, a central concern with these types of measures. As in the validation study by Ciobanu et al. [49], we also found strong correlations (from 0.59 to 0.73) between the three factors. These links were however slightly lower than expected. Due to the multicollinearity of predictors, we first chose to do a cluster analysis in order to showcase the different profile types which separated teachers into different patterns.

### 2.3. Data Analysis

The data collected through the online questionnaire were analyzed with SPSS (IBM Corp., Armonk, NY, USA). We found less than 1% of missing data overall on the questionnaires. No respondent has skipped enough items that this will bias the measures. Therefore, all participants were included in our analyses.

## 3. Results

Before highlighting profiles among the subjects in our sample, we compared the negative affectivity scores with the data from the validation of the questionnaire [49]. It should be noted that the reference sample (N = 1143) is composed of university students, and therefore of subjects who are clearly younger (mean age = 23.8) and who are generally not professionally integrated. However, these data (collected with the same questionnaire (also in French)) allowed us to highlight significant differences (one-sample *t*-test) in favour of the teachers in our sample (Table 1). We note in particular that the anxiety dimension, which scores relatively low among teachers, is very marked among students (no doubt due to their precarious situation and concerns about their future professional integration). It would be necessary to have other reference data in order to better position our sample.

The following step in our analyses was to develop groups of teachers with similar negative affectivity profiles. The various methods and their cluster analysis algorithms can lead to different conclusions depending on the sample size and the complexity of the model selected [56]. For this reason, the dynamic cloud method (K-means clustering) is often subject to discussion regarding the number of clusters to be retained [57]. That is why, as suggested by Milligan [58], we determined the optimal cluster structure by conducting a hierarchical analysis (using Euclidean distance and Ward’s method) first. This step leads to identify the optimal number of clusters for the second one. Then, we used K-means clustering to achieve clusters with the highest within-cluster similarity and the greatest between cluster variability.

**Figure 1:**
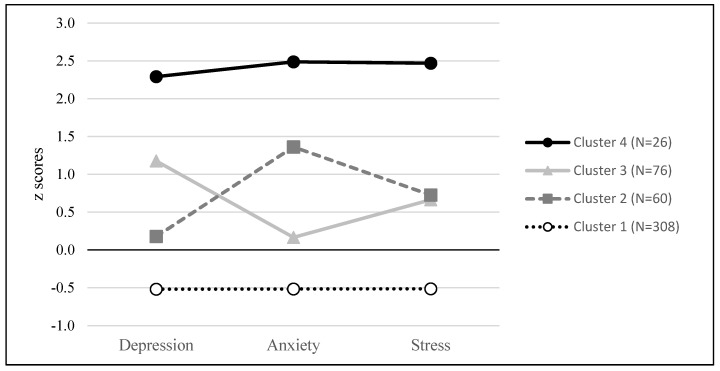
Final cluster among teachers.

Thus, we previously transformed the data of the three dimensions of negative affectivity into standardized values in order to avoid differential weighting of the dimensions in cluster analyses. Then, the hierarchical analysis revealed the presence of four distinct clusters. Finally, through K-means clustering, we were able to observe the grouping of teachers around these four very particular types (see Figure 1).

Two-thirds of our sample is in cluster 1 which includes teachers without particular negative affective traits. Scores for the three dimensions (depression, anxiety, and stress) are below average. Cluster 4 shows a profile at the other end of the spectrum from cluster 1. It has very few participants in it who all have strong negative affective traits—more than two standard deviations from our sample’s average on all three dimensions. In terms of the concrete implications of this result, we can be relatively satisfied that few teachers present a markedly negative profile. However, this statement ignores the fact that our sample is probably not totally representative of the population, since even if the response rate is high (for an online survey on a voluntary basis), we do not know the characteristics of those who did not respond. Thus, it is possible that teachers with a more pronounced professional malaise did not respond to our request. In terms of research, the size of this group is small. However, we focus more on the intermediate clusters. Thus, clusters 2 and 3 include participants with a medium tendency to feel stress (slightly above average) but who are markedly different on the other two dimensions (anxiety and depression).

Looking at the composition of these four clusters, we find that no differences are related to age (usually strongly correlated with years of experience). This factor, this time related to burnout, is controversial. While Friedman and Farber’s [59] findings indicate greater emotional exhaustion and depersonalization in younger teachers, more recent research (e.g., [60]) shows that burnout is independent of age or years of experience. With regards to gender, the male/female proportion is slightly different between clusters. However, while the proportion deviates the most from the baseline sample in cluster 4, it should be noted that this cluster consists of only 26 subjects, which does not allow for very strong conclusions.

**Figure 2:**
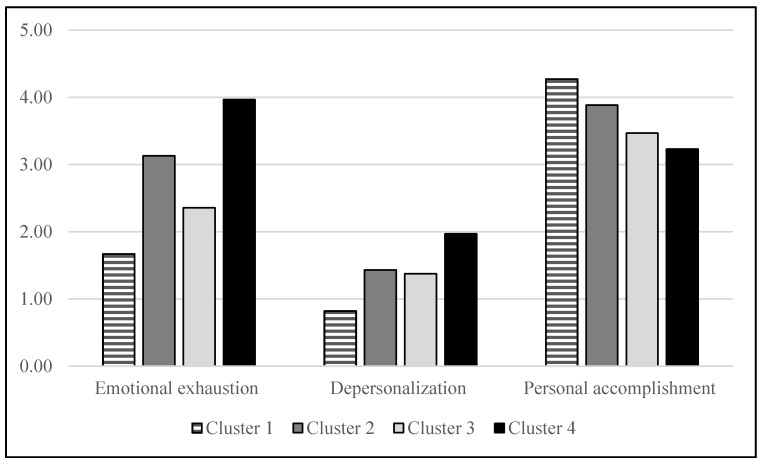
Burnout differences by clusters.

We sought to highlight the differences in scores in the three dimensions of burnout between these clusters (see Figure 2). Since the size of the groups is very different, we opted for Kruskal-Wallis tests. Indeed, the tests of homogeneity of variances carried out as a prerequisite for analyses of variances indicate biases, which leads us to use a non-parametric approach. Our analyses thus made it possible to highlight significant differences (see Figure 1) for emotional exhaustion (H(3) = 141.03; *p* < 0.01), for depersonalization (H(3) = 80.57; *p* < 0.01), and also for the decrease in personal accomplishment (H(3) = 116.60; *p* < 0.01).

Regarding pair comparisons (post hoc; significance values adjusted by the Bonferroni correction for multiple tests), for emotional exhaustion, all differences are significant, except for those between cluster 2 and cluster 4. For depersonalization, it is only cluster 1 that presents a score significantly different from the three other clusters. Finally, and for personal fulfillment, there are again significant differences between all clusters except this time between clusters 3 and 4. It should be noted that for this third dimension, the dispersion within the groups is clearly lower than that observed elsewhere. The descriptive statistics presented by the authors of the questionnaire [3], as well as in the French validation [54] report very similar distribution characteristics in terms of dispersion, but also scores that are in the high range of the scale.

In general, we can see that the difference between clusters 1 and 4 is systematically the most marked. It is also the one that is most obvious to understand. Indeed, these are contrasting groups with regard to the three dimensions of negative affectivity, and in particular with regard to the tendency to feel the presence of stressors more easily. Thus, these two groups have overall low vs. high levels of neuroticism and can be contrasted with the results of research that does not go into detail on the neuroticism subscales. These studies (in particular those considered in Swider and Zimmermann’s [24] meta-analysis, but also more recent publications (e.g., [41,42])) clearly underline the impact of neuroticism on subjects’ emotional exhaustion.

Cluster 1, which includes a very large number of subjects with the lowest presence of negative affectivity, has a clearly lower emotional exhaustion score than what is observed in the other groups. Similarly, in terms of depersonalization, this group shows a significantly lower level than the other groups with a floor effect in the distribution of scores. It is also the group for which personal fulfillment is the highest. Thus, whatever the dimension concerned, cluster 1 (notably due to its large number of employees) shows significantly less burnout than the other groups.

Cluster 4, on the other hand, systematically presents the least favorable pattern of scores. However, the scores taken separately in the three dimensions of burnout are not always significantly different from the intermediate clusters. For emotional exhaustion, the 26 subjects show (while having high scores) a more marked dispersion than in the other groups. This greater heterogeneity is also found for the other two dimensions, but in a mitigated manner. Unsurprisingly, the teachers who developed a more marked negative affectivity were also those who felt more burnt out. This result is fully consistent with the results of studies that highlight the links between negative affectivity and burnout in general (e.g., [27,41]).

Due to the presence of a small group with very high levels of negative affectivity and two thirds of the sample with clearly low scores, the correlation analyses do not make it possible to highlight what happens between these two extremes, which tends to mask individual differences and could lead to a simplistic approach to burnout in which only the vision of the combined scenario is visible [10]. Indeed, this author emphasizes that, in addition to the two pathways (emotional and cognitive), the combined scenario is often retained as the predominant model. This is why the most interesting differences concern those between the intermediate clusters (2 and 3), as they give us relevant information on the links between negative affectivity profiles and burnout. Indeed, these two groups present a very similar tendency to feel stress (with relatively low standard deviations), but they differ on the two other components. On the one hand, there are teachers who feel anxiety more strongly in their daily life (cluster 2). These are subjects who feel more emotionally exhausted since their score (without reaching that of cluster 4) presents a high level of burnout. We can suppose that it is a “pressure” pattern that would be responsible for the development of the burnout syndrome, with not only the presence of stress, but also a feeling of anxiety that would first lead the subject to feel a greater emotional exhaustion. The personality of these subjects could be similar to the “feeling” types proposed by Garden [61]. In line with Friedman’s theory [10], this would be an emotional scenario leading the teacher to feel unable to cope with the various stressors. In terms of personal accomplishment, however, these subjects show high values, significantly different from clusters 3 and 4.

Another group (cluster 3) of relatively similar size showed an inverse pattern with higher depression scores (on the negative affect scale) than group 2, but lower anxiety scores. With a lower level of emotional exhaustion (but which cannot be considered meaningless), this group is distinguished above all by a much lower degree of personal accomplishment than for cluster 2. This greater tendency to feel depressed leads these teachers to experience a lower level of personal accomplishment. This time, it is more a question of a “depression” pattern that would be implemented with a predominant impact on the self-esteem of people who are initially less intuitive and who question themselves more (“thinking” type according to Garden [61]). The cognitive scenario [10] would then be more present in this category of teachers, who would be more likely to engage in ruminations and highly depreciatory self-evaluations.

## 4. Limitations

There are several limitations to discuss with respect to our results. First, we observe a relatively small number of subjects in clusters with more pronounced negative affectivity traits. Indeed, only one third of our total sample is distributed in clusters 2, 3, and 4. While this finding is quite pleasing in epidemiological terms, the findings (and in particular the analysis of the differences between the two intermediate clusters) are based on a limited number of teachers. Larger scale research should be conducted, in particular to verify whether the differences found in our sample are found on a larger scale.

The second limitation is the possible bias associated with self-reported questionnaires with volunteer subjects. Indeed, we measured the negative affectivity traits as well as the burnout measures through self-reported measures in which subjectivity is present. In this sense, it is likely that individuals with a stronger tendency to negative affective feelings also have a natural tendency to view the burnout items more pessimistically. Such a bias could explain the very marked differences between the most extreme groups (clusters 1 and 4).

Thirdly, the method chosen in this study does not allow for any causal link to be inferred between personality and burnout. Although personality factors develop early in life and are relatively stable, one would need to do longitudinal studies to see how things evolve over time to draw causal relationships between personality and burnout.

Finally, our research does not take into account measures concerning stress factors experienced in the work context and in the personal environment. Similarly, the presence and satisfaction with possible social support in their work context (a buffer factor in the development of burnout [62]) is not measured. Such variables could be able to reinforce, respectively mitigate, the impact of negative affectivity on the development of burnout.

## 5. Conclusions

While the links between personality factors (in particular neuroticism) and burnout are well documented in the scientific literature [24] considering different facets of negative affectivity seems to be an interesting avenue to highlight finer personality differences and thus better understand the development of burnout.

Among the four profiles that emerge from our analyses, we note that two clusters correspond to personalities marked by a low, respectively, high, negative affectivity, which is linked to the three dimensions of burnout. These initial results thus confirm the importance of teachers’ personalities in their reactions to the many stressors present in their professional context. The two intermediate profiles (one marked by the anxiety dimension and the other characterized by the depression dimension) make it possible to make a distinction that can help us better understand the forms of burnout. Thus, our results support the Multiple Pathway to Burnout (MPB) model proposed by Friedman [10]. Indeed, the tendency to experience a fair amount of anxious affect in stressful situations can be likened to the emotional scenario of MPB, or the “feeling” type [61]. These teachers are in a pressure dynamic where the stressors (and even their apprehension) lead to a marked emotional exhaustion. The other intermediate profile is characterized by self-deprecation, anhedonia and other depressive symptoms. It corresponds to the cognitive scenario of the MPB, or the “thinking” type [61]. Teachers with these profiles are then in a depression logic which initially has a more marked impact on the reduction of personal accomplishment.

Thus, depending on the personality profile, the dimensions of burnout are not affected in the same way. These intermediate groups (which we can imagine, according to the MPB [10], are in a phase of emergence of stress-induced experiences) the process of burnout development has not yet affected depersonalization (whose score is moderate). These results therefore support the idea that depersonalization (including cynicism) is a dysfunctional coping response [61,63]. However, our research does not employ a longitudinal research design that would confirm the sequence. It would be interesting, albeit ethically problematic, to follow the evolution of these two intermediate groups in order to examine the extent to which the other dimensions of burnout may then be affected.

Our research focused on a single personality factor (neuroticism). Although this dimension has a dominant influence on burnout [31], other personality factors are also likely to play a significant role in the development of this syndrome, in particular, the dimensions agreeableness and extraversion which can be considered as the interpersonal dimensions of personality [64]. It would therefore be interesting to take into consideration the various facets of these two dimensions in order to assess more finely the extent to which certain personality profiles are more likely to experience burnout. In particular, we could expect a greater impact on depersonalization and thus give a punctual argument to the model proposed by Golembiewski and Muzenrider [8] which suggests that the burnout process starts with a significant social withdrawal.

In his symposium address, Gendron [65] notes that teaching is a social and affective act and that teachers’ well-being impacts the well-being, satisfaction, motivation, health and performance of their students. Roffey [66] reminds us that teacher attrition is an issue in many countries. Therefore, looking at how one can improve the lives and well-being of teachers may help stop them from changing careers.

Knowing that teachers’ well-being impacts that of students [12,66], it becomes even more critical to address burnout in order to preserve teachers from exhaustion and their students from related stress. Furthermore, as personality is also related to emotional competencies [67], taking into account how teachers use such competencies (to manage not only their own emotions but also those of their students) would not only allow for more comprehensive profiles to be drawn up but also for concrete avenues to be explored in order to decrease the risk of burnout and to promote a more serene classroom climate for better emotional outcomes for students. Indeed, since personality factors are considered relatively stable over time [32], acting on emotional competencies could be an approach capable of reducing the impact of stress in the classroom (also among students) and avoiding the development of burnout, particularly among novice teachers facing many uncertainties leading to negative affects [68]. Certain emotional competencies reduce the risk of burnout [15], in particular by improving the social climate and the functioning of the classroom in general [69]. By profiling teachers’ personalities, particularly with regard to negative affectivity, it would be possible to determine which emotional competencies should be developed as a priority in order to deal with the development of burnout in a targeted manner. However, ethically speaking, it would be difficult to systematically evaluate all teachers’ personalities. In practice, assessments are made and solutions offered once a person shows their first symptoms and/or is being taken into care. Those assessments and solutions take into consideration other contextual factors not included in this research.

When it comes to intervention (more so than prevention) it is necessary to factor in individual needs. Although some emotional competencies reduce the risk of burnout [15], in particular by improving the social climate and the functioning of the classroom in general [69], some emotional competencies (e.g., an acute perception of internal bodily affect indicators [70]) can also accentuate stressors’ negative impact. Better knowledge of teachers’ ways of working on a personal and interpersonal level when facing stress should allow for a more efficient way of helping them. The work that can be conducted by individuals, on emotional openness for example, can bring about better efficiency of targeted interventions since individual characteristics play an important role in a therapeutic setting [71]. Understanding an individual’s functioning is therefore an important step in his or her care.

## Figures and Tables

**Table 1:** Descriptives and comparisons between the subscales of negative affectivity of the present study and the validation sample [49].

	Present Study M (SD)	Ciobanu et al. (2018) M (SD)	Difference
Depression	7.43 (7.77)	8.25 (8.07)	t_(469)_ = −2.30, *p* < 0.05
Anxiety	3.91 (4.98)	8.05 (8.07)	t_(469)_ = −18.01, *p* < 0.01
Stress	11.50 (8.30)	13.18 (8.26)	t_(469)_ = −4.38, *p* < 0.01

## Data Availability

Participants were guaranteed anonymity and the non-disclosure of individual data. Due to the nature of the data collected, it may be possible for people who know the teaching population to identify participants. For this reason, the data is not made publicly available.
